# Nanoscale Nitrogen Doping in Silicon by Self-Assembled Monolayers

**DOI:** 10.1038/srep12641

**Published:** 2015-07-31

**Authors:** Bin Guan, Hamidreza Siampour, Zhao Fan, Shun Wang, Xiang Yang Kong, Abdelmadjid Mesli, Jian Zhang, Yaping Dan

**Affiliations:** 1 University of Michigan - Shanghai Jiao Tong University Joint Institute; 2 Key Laboratory of Artificial Structures and Quantum Control (Ministry of Education), Department of Physics and Astronomy; 3 School of Materials Science and Engineering, Shanghai Jiao Tong University, Shanghai, 200040, China; 4 Institut Matériaux Microélectronique Nanosciences de Provence, UMR 6242 CNRS, Université Aix-Marseille, 13397 Marseille Cedex 20, France; 5 Faculty of Medicine, Shanghai Jiao Tong University, Shanghai, 200040, China

## Abstract

This Report presents a nitrogen-doping method by chemically forming self-assembled monolayers on silicon. Van der Pauw technique, secondary-ion mass spectroscopy and low temperature Hall effect measurements are employed to characterize the nitrogen dopants. The experimental data show that the diffusion coefficient of nitrogen dopants is 3.66 × 10^−15^ cm^2^ s^−1^, 2 orders magnitude lower than that of phosphorus dopants in silicon. It is found that less than 1% of nitrogen dopants exhibit electrical activity. The analysis of Hall effect data at low temperatures indicates that the donor energy level for nitrogen dopants is located at 189 meV below the conduction band, consistent with the literature value.

The successful development of the complementary metal-oxide-semiconductor (CMOS) technology in the past decades has generated a huge impact on the human society by offering devices with higher performances at lower cost^1^. The main driving force of this development is the constant downscaling of CMOS transistors, which, however, has become increasingly difficult. One of the main issues is the short channel effect that the transistor gate loses control as the size becomes smaller, resulting in malfunctions. Currently, the CMOS industry solves this issue by using a 3-dimensional “FIN” structure that allows the gate grabbing around the channel to enhance its control^2^. This solution unfortunately increases the complexity of the fabrication processes. The cost per device increases instead of continuously decreasing as Moore’s Law predicts^3^.

A second solution to alleviate the short channel effect is to form ultra-shallow junctions in the source and drain regions of the transistor. This is one of the main motivations of “gold rush” for developing transistors based on the 2-dimensional atomic monolayers such as graphene and MoS_2_, which will facilitate the formation of atomically thin junctions^4^,^5^,^6^. Silicon, as the most commonly used substrate in CMOS industry, is unlikely to be replaced by any new materials in the foreseeable future^7^. With silicon as the substrate, it has been difficult to form ultra-shallow junctions on the silicon surface by the traditional ion implantation doping process. This is because the physical bombardment of accelerated ions turns the single-crystalline silicon surface (about 10 nm thick) into low quality amorphous silicon, resulting in significant degradation in the device performances^8^. In recent years, doping by self-assembled monolayers (SAMs) has proven to be a mild and controllable doping process^9^,^10^. The process consists of chemical assembly of dopant-containing molecules on silicon surfaces and subsequent thermal treatment that drives dopants to diffuse into the substrate. To form ultra-shallow junctions, spike thermal treatment is often required for this process^11^. A sub-10 nm junction can be formed for fast diffusing dopants like phosphorus by limiting the annealing time to as short as a few seconds. However, a very short pulse of high temperature might lead to problems such as low reproducibility. It is more reliable to form ultra-shallow junctions using dopants with low thermal diffusion coefficient, because such dopants will allow extended periods of annealing time. Nitrogen, similar to phosphorus, is also a group V element acting as a donor-type dopant in silicon. Previous research on nitrogen diffusion in silicon has showed that nitrogen dopants introduced by implantation and rapid thermal annealing have low diffusivity^12^,^13^. Nitrogen doping by SAMs can potentially form ultra-shallow or even atomically thin junctions in silicon. It thus may provide a better solution to the short channel effect and allow for the continuous downscaling of the CMOS technology.

In this Report we investigate the properties of nitrogen doping by SAMs that carry doping elements such as phosphorus and nitrogen. The chemical composition of the SAMs and the doped silicon substrate were examined using X-ray photoelectron spectroscopy (XPS) and secondary-ion mass spectrometry (SIMS). Van der Pauw and Hall effect measurements were used to characterize the electronic properties of the dopants. We demonstrate the successful doping of nitrogen and phosphorus in intrinsic silicon wafer, extract the diffusion coefficients for both dopants and estimate the donor energy levels in the monolayer doped silicon samples.

## Results and Discussions

**Figure 1:**
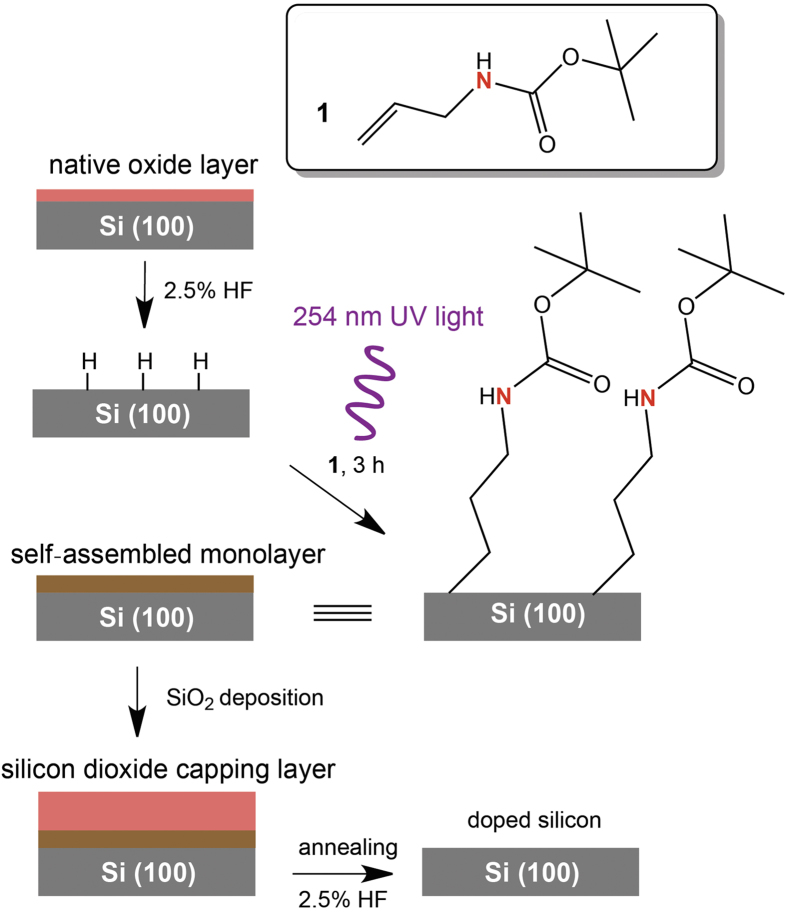
Schematic illustration of surface modification (SAMs formation) and monolayer doping on silicon.

**Figure 2:**
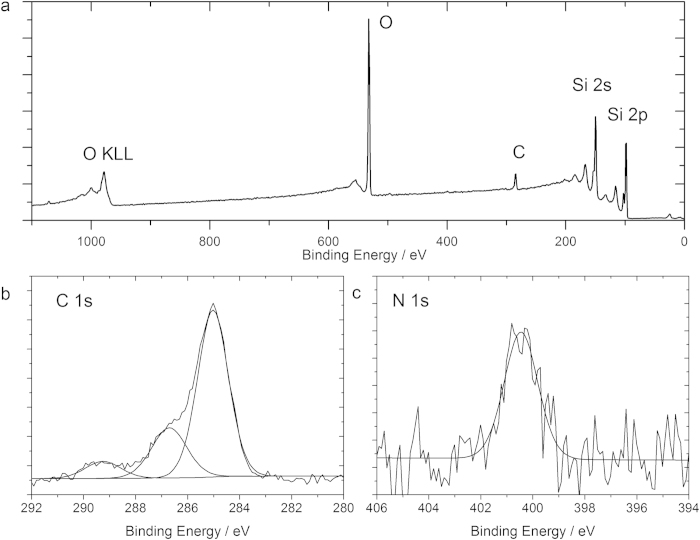
(**a**) XPS survey spectra of the alkene species **1** monolayer on silicon. (**b**) Narrow scan of the C 1s region. (**c**) Narrow scan of the N 1s region.

The preparation of SAMs on silicon followed the so-called hydrosilylation strategy, as shown in Fig. 1. The hydrosilylation process on silicon, first reported by Linford and Chidsey^14^,^15^, refers to the addition of silicon hydride onto an unsaturated carbon-to-carbon bond. It has been considered the most robust method for producing covalent Si−C bound organic monolayers on silicon^16^,^17^,^18^. Hydride-terminated surfaces were reacted with a nitrogen-containing alkene species **1** to form a silicon-carbon bound monolayer under UV light radiation. The strategies to modify the silicon surfaces with other molecules are shown in the supplementary Fig. S1. Figure 2 shows XPS spectra acquired on the silicon surface with alkene species **1** modification. The survey spectrum indicates the presence of Si, C and O, which is in good agreement with the presence of an organic monolayer on the silicon substrate. The oxygen 1s emission at 532 eV is ascribed to oxygen from the monolayer, as well as adventitiously adsorbed oxygen. Oxygen does not usually give reliable signals in XPS due to the contamination by the ambient atmosphere. However, both carbon and nitrogen can provide useful information on the nature of surface-bound species. The narrow scan of the C 1s region (Fig. 2b) shows a broad band at 285 eV (1.4 eV FWHM), attributed to aliphatic carbon-carbon (C−C) bonding. The side shoulder of the C 1s band at 286.5 eV (1.6 eV FWHM) is assigned to carbon-oxygen (C−O) and carbon-nitrogen (C−N) bonds from the molecule **1**. The peak at 289 eV (1.6 eV FWHM) accounts for the carbonyl (C=O) in the terminal group. These observed binding energies are consistent with those reported for carbon species on Si(100) surfaces^19^. Moreover, the representative peak area ratio of C−C to C−O/C−N shows an experimental value of 2.88:1, which is in agreement with those predicted by the stoichiometry of the atoms on the putative surface product (2.5:1). The narrow scan spectrum of N 1s in Fig. 2c displays one component at 400.5 eV (1.6 eV FWHM), corresponding to the nitrogen atom at *tert*-Butoxycarbonyl (*t*-Boc) group of alkene **1**. Therefore, it is suggested that an alkene species **1** monolayer is formed on the silicon surface. We also employed XPS to analyse other SAMs formed on silicon according to the scheme in supplementary Fig. S1. Some oxides have been detected on the modified silicon surface (supplementary Fig. S2), although it was reported that an oxide-free surface after hydrosilylation on Si(100) could be achieved^20^,^21^,^22^. The data and discussions can be found in the supplementary Fig. S2 and Fig. S3.

The monolayer modified samples were then coated with a 20-nm thick layer of silicon dioxide by atomic layer deposition (ALD) and subsequently annealed at 1050 °C for 2 min (Fig. 1). To evaluate the nitrogen doping process, Van der Pauw technique was employed to measure the sheet resistance of the silicon samples at room temperature. Table 1 lists the experimental results of the silicon samples with and without nitrogen doping, as well as the sample with both nitrogen and phosphorus doping (for the I-V curves, refer to the supplementary Fig. S4). The sheet resistance of the original undoped silicon substrate was measured to be 143 ± 21 kΩ. A piece of silicon wafer labeled as “blank sample” was selected to undergo all the capping and annealing processes except the SAM formation. The sample shows a sheet resistance as high as 121 ± 13 kΩ, indicating negligible contamination from the capping SiO_2_ and the ambient environment of the annealing chamber. A control sample with the absence of doping atoms was prepared to test the possible influence of C, O and H to the doping process. No significant change was observed from the control sample with only C monolayer modification (see the scheme in Supplementary Fig. S1). This means that C and O atoms, when sitting at their natural sites, substitutional for carbon and interstitial for oxygen, are electrically inactive in silicon. The samples that were modified with the monolayers containing N or both N and P (Supplementary Fig. S1) experience a significant drop in the sheet resistance. Clearly, the drop in the sheet resistance is solely contributed by N or both N and P that are carried by the SAMs. Electrostatic force microscopy (EFM) was also applied on the monolayer doped silicon samples to obtain the static charge profile across the samples. The EFM images in Supplementary Fig. S5 showed no clear aggregation of dopants across the samples, suggesting a relatively uniform doping.

**Figure 3:**
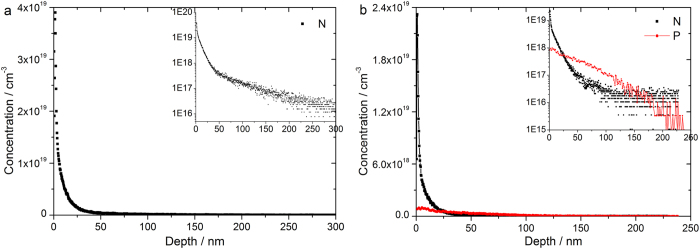
Nitrogen and phosphorus doping profiles in sample #1 (a) and sample #2 (b). Inset graphs show the nitrogen and phosphorus concentration in log scale.

To access to the doping profiles, the doped samples were analysed by SIMS, as shown in Fig. 3. In sample #1 with only nitrogen as dopant, the dopant concentration drops rapidly from ~4 × 10^19^ cm^−3^ near the surface to ~10^17^ cm^−3^ within a distance of 100 nm. In sample #2 with both nitrogen and phosphorus doping, nitrogen shows the same trend of diffusion residing in the top 100 nm of the substrate. Phosphorus dopants, however, diffuse deeper than nitrogen to 200 nm below the interface, leading to a lower dopant concentration near the surface than nitrogen as required by the mass conservation. These dopants are carried by the SAMs, the nature of which limits the total number of initial dopants on the surface. After high temperature annealing, the dopants will follow the limited-source diffusion process, which is governed by the equation below:


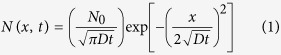


where *N*_0_ is the initial surface concentration, *x* the diffusion distance, *t* the annealing time and *D* the diffusion constant which is temperature dependent.

By fitting equation (1) into the curves in Fig. 3, we find that the diffusion coefficient for nitrogen is 3.66 × 10^−15^ cm^2^ s^−1^ in sample #1 and 1.39 × 10^−14^ cm^2^ s^−1^ in sample #2. The diffusion coefficient for phosphorus in sample #2 is 1.69 × 10^−13^ cm^2^ s^−1^ which is comparable to the widely observed value for phosphorus in silicon^23^,^24^, but two orders of magnitude larger than that of nitrogen in intrinsic silicon (sample #1). The diffusion of nitrogen follows a complex behavior in which defects distribution and some other factors during annealing process play key roles^25^,^26^. It is likely that the diffusion of nitrogen dopants can be enhanced by interaction with the faster diffusing phosphorus dopants. This is in line with the aforementioned values showing that the diffusion coefficient of nitrogen in the phosphorus-contained silicon (sample #2) is relatively larger than that in sample #1 without phosphorus.

High temperature annealing aims at accomplishing two steps: (i) driving the dopant atoms from the surface towards the bulk; (ii) activating them electrically by allowing doping atoms to find substitutional sites in the lattice. The first step is verified by SIMS, while the second is measured by Van der Pauw technique and Hall effect measurement. The comparison of the two approaches allows for informing about the activated versus total fraction of introduced dopants.

From the SIMS data in Fig. 3, we can calculate the total amount of dopant atoms that diffuse from the surface into the bulk. In sample #1 the average nitrogen dopant concentration (*n*_*d*_) per unit area is 2.36 × 10^13^ cm^−2^, and the average nitrogen and phosphorus dopant concentration per unit area in sample #2 are 9.35 × 10^12^ cm^−2^ and 4.3×10^12^ cm^−2^, respectively. The amount of nitrogen diffusing into substrate in sample #2 outnumbers that of phosphorus by a factor of around 2, which is consistent with the fact that ~65% of phosphate molecules are coupled onto nitrogen modified surface (see supplementary Fig. S3). If the nitrogen dopants were 100% electrically active in sample #1, the sheet resistance of the sample would be around 188 Ω. This is lower by a factor of about 200 than the measured sheet resistance listed in Table 1, indicating that the nitrogen dopants were not fully electrically activated. The activation rate can be estimated by the ratio 188/35517 = 0.53%. Similar conclusions can be reached for sample #2. However, it is not straightforward to find the exact activation rate of the dopants from the resistivity measurements, because the carrier mobility is dependent on the concentration of the activated dopants that are highly non-uniform in our case. We therefore turn to the Hall effect measurements that can directly provide the carrier concentration without the information of carrier mobility.

For Hall effect measurements, the Hall voltage (*V*_*H*_) is governed by the following equation:


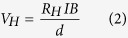


where *R*_*H*_ is the Hall coefficient which is equal to 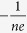 for n-type semiconductors with the electron concentration *n* and unit charge *e*, *I* the current flow, *B* the magnetic field, and *d* the thickness of the sample.

Considering that the electron concentration is non-uniform from the surface to the bulk, Hall resistance can be written in the following expression in which *n*_*c*_ is the electron concentration per unit area.


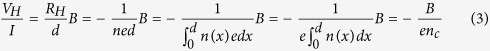


**Figure 4:**
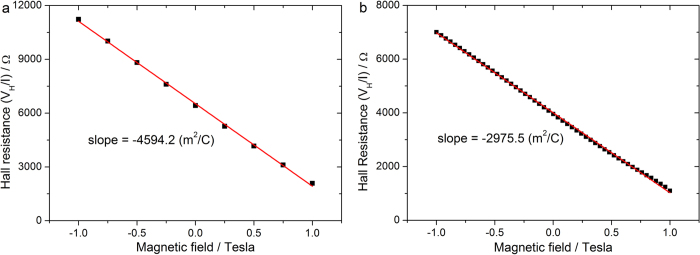
Hall resistance dependence on the magnetic field at 300 K for sample #1 (a) and sample #2 (b).

Figure 4 displays the Hall effect measurement results on both samples in Hall bar geometry at 300 K. The Hall resistance is linearly correlated with the magnetic field. It is worth pointing out that the measured value of Hall resistance is influenced by the longitudinal misalignment and the thermoelectric voltage between the two Hall contacts. Hence in Fig. 4 Hall resistance shows a background number when the magnetic field is zero, which however does not influence the extraction of the free carrier concentration. From the slope of the linear correlation, the free electron concentration per unit area (*n*_*c*_) is extracted to be 1.36 × 10^11^ cm^−2^ in sample #1 and 2.1×10^11^ cm^−2^ in sample #2. Compared to the SIMS data, we find that the activation rate for N in sample #1 is only 0.58% (a value very close to the crude estimation 0.53% given above), and the average activation rate for N and P in sample #2 is approximately 1.54%. Less than 1% activation rate of nitrogen in our case is consistent with the research in 1970s^27^, as the fraction of substitutional nitrogen is small. It is known that phosphorus implanted silicon has a high degree of electrical activity compared to nitrogen. However, it was reported that the presence of nitrogen could retard phosphorus activation by forming inactive complexes of phosphorus and nitrogen atoms at a ratio of 1:1^28^. That might be the cause of low activation rate for sample #2. It also has been shown previously that P dopants carried by SAMs (not containing N) have an ionization rate over 90%^9^. We conclude that the low activation rate is not intrinsic to this method, but highly dependent on other impurities in the substrate. For example, it was shown that the activation rate of N dopants by ion implantation could be significantly increased in a boron doped silicon substrate^29^. Those boron atoms residing at the lattice sites of silicon can be displaced by silicon self-interstitials and later nitrogen atoms can fill the vacancies to render nitrogen donors. As a result, the activation rate of nitrogen could be significantly improved. The work using boron-doped silicon substrates to increase the electrical activation of nitrogen dopants by the SAM method is under investigation.

**Figure 5:**
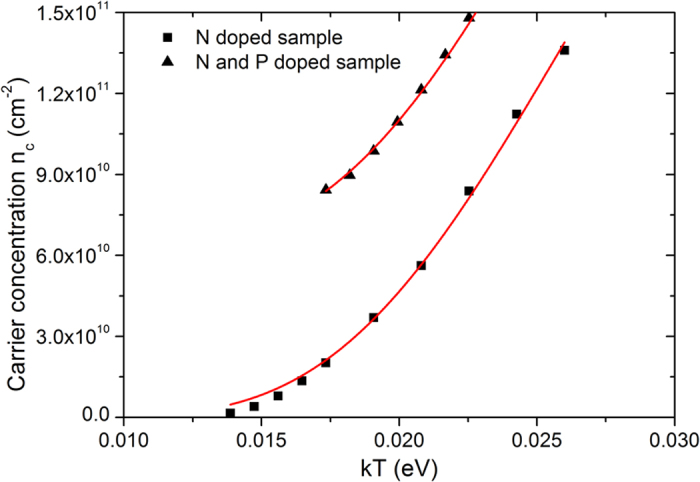
Temperature dependence of the average free electron concentration for sample #1 and #2.

To further investigate the properties of dopants, we performed low temperature Hall effect measurements, from which the activation energy of the dopants can be found. We first extracted the average free electron concentrations (*n*_*c*_) at different temperatures from the curves of Hall resistance dependence on magnetic field for both N doped sample and N&P doped sample (see the supplementary Table S1), which are similar to those in Fig. 4 except that they show some nonlinearity at low temperatures. The details are illustrated in the supplementary Fig. S6 and S7. Then we plot the surface concentration of *n*_*c*_ as a function of *kT* in Fig. 5, where *k* is the Boltzmann constant and *T* is the absolute temperature. The dopant distribution is highly non-uniform within the thin-doped layer near the surface. But the average concentration of electrons (*n*_*c*_), holes (*p*_*c*_) and ionized dopants (*n*_*D*_^*+*^) will always remain charge neutral. On the assumption that the holes are negligibly small in concentration compared to the electrons, which is true in our case, we can derive the electron concentration *n*_*c*_ that is correlated with the temperature as the following expression (see the supplementary materials for details):


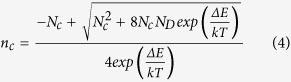


where *N*_*c*_ is the effective density of states function which is defined as 
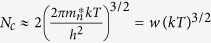
 with *w* being the constant related to the band structure of the semiconductor, *N*_*D*_ the concentration of donors, and *ΔE* the activation energy which is equal to (*E*_*c*_ *−* *E*_*d*_) with *E*_*c*_ and *E*_*d*_ being the conductance band edge and the donor energy level, respectively.

By fitting equation (4) into the curve in Fig. 5 for N doped sample, we can obtain the activation energy of the nitrogen dopants as 189 meV and some other parameters as listed in Table 2. In the N and P doped sample, there are two types of donors to generate free electrons. As phosphorus dopants in silicon have much lower ionization energy than nitrogen, which is ~44 meV^30^, P atoms that replace Si lattice remain complete ionization within the temperature range of our measurements. For the N and P sample, equation (4) can be rewritten as


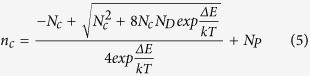


where all the parameters have the same meaning as in equation (4) except that *N*_*P*_ is the average concentration of completely ionized P donors.

The fitting results of equation (5) are also displayed in Fig. 5 and Table 2. The concentration of N donors (*N*_*D*_) and the activation energy for N atoms (*ΔE*) derived from the two equations show similar results, ~3.4×10^11^ cm^−2^ and ~189 meV, respectively. The activation energy we obtained here shows a deep energy level existing for N dopants in Si, which is in good agreement with the literature^31^.

According to the fitting values in Table 2, the concentration of N donors in N-doped sample is 2-order magnitude lower than the total nitrogen concentration detected by SIMS, indicating that only about 1% of the nitrogen atoms diffusing into silicon bulk have substituted the Si lattice. This is again consistent with the report that more than 95% implanted nitrogen in silicon is trapped on some non-substituional sites or defects in the form of molecular N_2_, silicon nitride precipitates or randomly distributed atomic nitrogen^32^. Similarly, only 1.4% of phosphorus atoms have substituted the Si lattice. The rest of P atoms may form the inactive nitrogen-phosphorus complexes with N atoms as mentioned previously^28^.

In conclusion, we have successfully demonstrated that the nitrogen-containing SAMs on intrinsic silicon surface leads to nitrogen doping in the substrate with the assistance of high temperature annealing. The activation rate and diffusion coefficient of nitrogen dopants were found to be consistent with literature values. As both parameters are related to defects such as silicon interstitials in the substrate, nitrogen doping with a higher activation rate might be achieved on p-type Si substrates. Based on the Van der Pauw and Hall effect measurements, a deep donor energy level at 189 meV below the conduction band was found for N doped silicon, which is associated with substitutional nitrogen. In future, DLTS (deep level transient spectroscopy) measurements will be employed to obtain further information about dopants and defects in nitrogen-doped silicon.

## Methods

### Wafer cleaning

Float Zone single-side polished silicon wafers, (100) surface orientation ((100) ± 0.05°), 500 ± 25 μm thick, 9000–15000 Ω cm in resistivity, were cleaved into 1 cm by 1 cm pieces and cleaned with acetone and ethanol of CMOS grade in a sonication bath for 5 min, respectively. After rinsed with DI water, the Si samples were immersed in “piranha solution” (98% H_2_SO_4_:30% H_2_O_2_, 3:1 (v/v)) for 30 minutes at 90 °C, followed by rinsing with DI water again. The wafers were then etched in 2.5% hydrofluoric acid (HF) solution for 90 seconds to remove the oxide layer and rinsed with DI water, followed by drying under N_2_ stream.

### UV-mediated hydrosilylation and surface functionalization

The freshly etched Si(100) samples were immediately placed in the reaction tube, covered with 30–50 μL of 50 mg/mL tert-Butyl N-Allylcarbamate (TCI America, >98%) methanol solution and were exposed to the irradiation of a 254 nm UV lamp (0.36 mW/cm^2^) in an inert nitrogen atmosphere for 3 h, affording sample #1. Control experiment was conducted on freshly prepared Si samples with neat undecylenic acid (J&K Scientific Ltd., >97%) under 254 nm UV radiation overnight. The samples were then rinsed with copious dichloromethane and ethanol, and dried under nitrogen gas. To obtain samples with phosphorus functionalization (sample #2, see Supplementary Fig. S1), sample #1 was subjected to 25% trifluoroacetic acid (TFA) in dichloromethane for 3 h, followed by a 10 min rinse in 10% ammonium hydroxide (NH_4_OH) to form the primary amine terminated surface. The surface was immersed in an ethanol solution of bifunctinal crosslinker dicyclohexylcarbodiimide (DCC, 40 mΜ, Aldrich) and mono-Dodecyl phosphate (5 mΜ, Aldrich) for 6 h, affording sample #2.

### Silicon dioxide deposition and thermal annealing

SiO_2_ films were grown by atomic layer deposition on Si samples with tris(dimethylamino)silane (TDMAS) and ozone as precursors. Thermal annealing at 1050 °C for 120 s, with a ramp temperature of 100 °C/min, starting from 800 °C, was performed in an argon environment in a tube furnace (Thermo scientific Lindberg/Blue, USA). After annealing, the Si samples were immersed again in 2.5% HF solution to remove SiO_2_ film on the surfaces.

### Surface Characterization

X-ray photoelectron spectroscopy (XPS) was carried on a Kratos AXIS UltraDLD spectrometer with a monochromated Al Kα source (1486.6 eV), a hybrid magnification mode analyser and a multichannel detector at a takeoff angle of 90° from the plane of the sample surface. Analysis chamber pressure is less than 5 × 10^−9^ Torr. All energies are reported as binding energies in eV and referenced to the C 1s signal (corrected to 285.0 eV) for aliphatic carbon on the analysed sample surface. Survey scans were carried out selecting 250 ms dwell time and analyser pass energy of 160 eV. High-resolution scans were run with 0.1 eV step size, dwell time of 100 ms and the analyser pass energy set to 40 eV. After background subtraction using the Shirley routine, XPS spectra were fitted with a convolution of Lorentzian and Gaussian profiles by using software Casa XPS. Secondary-ion Mass Spectroscopy (SIMS) was conducted to obtain dopant profile at the top 300 nm of substrate at Evans Analytical Group, NJ, USA. EFM experiments were performed on a Nanonavi E-Sweep system (Seiko, Japan) operated in tapping mode.

### Electrical Characterization

The metal contacts on silicon for electrical measurements were realized by evaporating 200-nm aluminum or chromium/gold films in a thermal evaporation system (Angstrom Engineering, Canada). Van der Pauw measurements were performed on square-shaped samples on which the metal contacts are exactly located at the four corners. The custom-made probe station is equipped with four solid tungsten probe tips (the tip size < 1 μm). Keithley 2400 sourcemeter units and a custom-written Labview script were employed to generate and collect current/voltage data. Hall effect measurements were performed on samples in Hall bar geometry in a Physical Property Measurement System (PPMS, Quantum Design, USA).

## Additional Information

**How to cite this article**: Guan, B. *et al.* Nanoscale Nitrogen Doping in Silicon by Self-Assembled Monolayers. *Sci. Rep.* **5**, 12641; doi: 10.1038/srep12641 (2015).

## Supplementary Material

Supplementary Information

## Figures and Tables

**Table 1:**
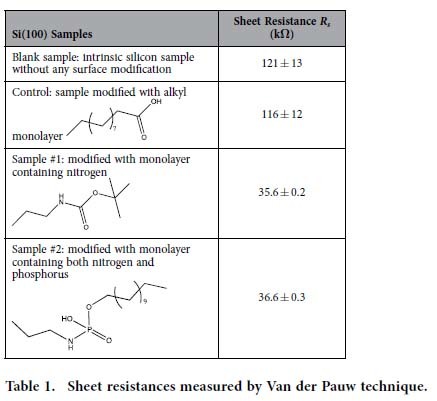
Sheet resistances measured by Van der Pauw technique.

**Table 2:** Fitting results of equations (4) and (5) to the plots of *n*_*c*_ as the function of *kT*.

**Fitting results**	**Parameter**	**Value**	**Standard error**
N doped sample (sample #1), fitted with equation (4)	*N*_*D*_ (×10^9^ cm^−2^)	334	30
	*ΔE* (eV)	0.189	0.003
N and P dope sample (sample #2), fitted with equation (5)	*N*_*D*_ (×10^9^ cm^−2^)	348	47
	*ΔE* (eV)	0.188	0.007
	*N*_*P*_ (×10^9^ cm^−2^)	61.8	4.6
