# Supplementary material for: Nanoscale Nitrogen Doping in Silicon by Self-Assembled Monolayers
Source: Sci Rep. 2015 Jul 31;5:12641. doi: 10.1038/srep12641 (PMC4521158; doi:10.1038/srep12641)
Supplement: Supplementary Information [file srep12641-s1.pdf]

Supporting Information for

Nanoscale Nitrogen Doping in Silicon by Self-Assembled Monolayers

Bin Guan [1], Hamidreza Siampour [1], Zhao Fan [1], Shun Wang[2], Xiangyang Kong[3], Abdelmadjid Mesli [4], Jian Zhang [5*], Yaping Dan [1*]

[1] University of Michigan - Shanghai Jiao Tong University Joint Institute,

[2]Key Laboratory of Artificial Structures and Quantum Control (Ministry of Education), Department of Physics and Astronomy,

[3]School of Materials Science and Engineering,

Shanghai Jiao Tong University, Shanghai, 200040, China.

[4] Institut Matériaux Microélectronique Nanosciences de Provence, UMR 6242 CNRS, Université Aix-Marseille, 13397 Marseille Cedex 20, France

[5] Faculty of Medicine,

Shanghai Jiao Tong University, Shanghai, 200040, China.

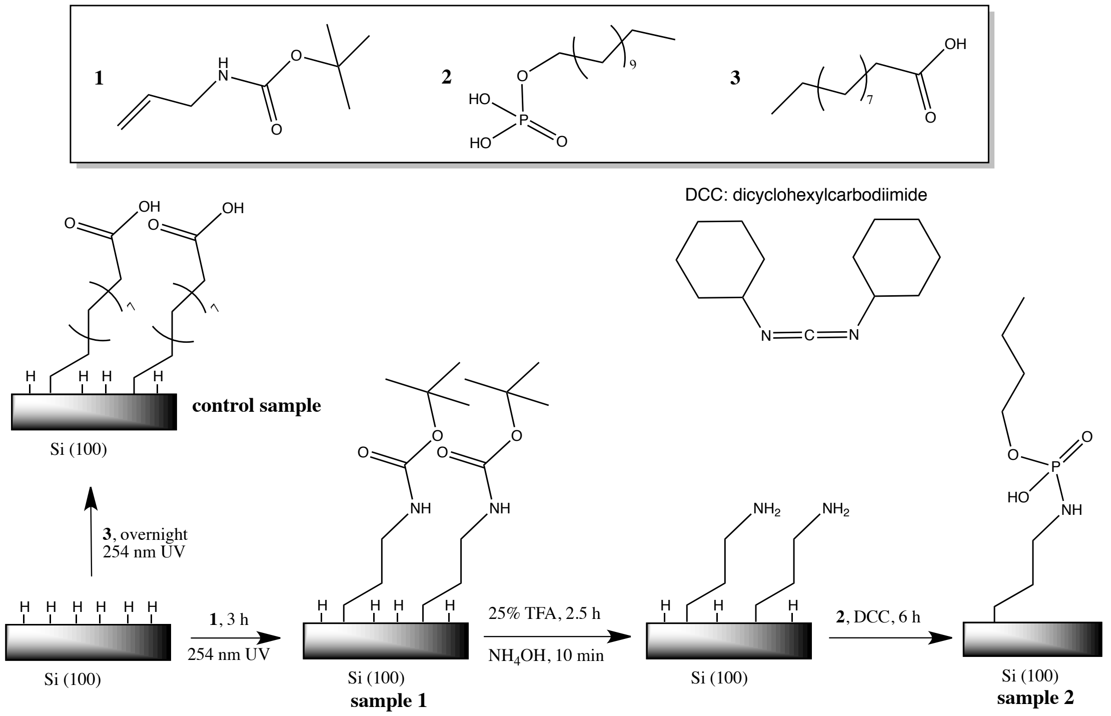

Figure S1. Surface modification routes for preparing monolayers without nitrogen (control sample) and with both nitrogen and phosphorus functionalization (sample #1 and #2). The freshly etched hydride-terminated Si (100) samples were immersed in neat deoxygenated undecylenic acid **3** and were reacted under the irradiation of a 254 nm UV lamp in an inert nitrogen atmosphere for over 16 h. The samples were then rinsed with copious dichloromethane and ethanol, and dried under argon gas, yielding control sample. Sample #2 was prepared with further modification on alkene species **1** modified sample #1. Following by amine deprotection in 25% trifluoroacetic acid, the sample was derivatized with phosphate **2** in the presence of cross-linker DCC, providing monolayers containing both nitrogen and phosphorus atoms.

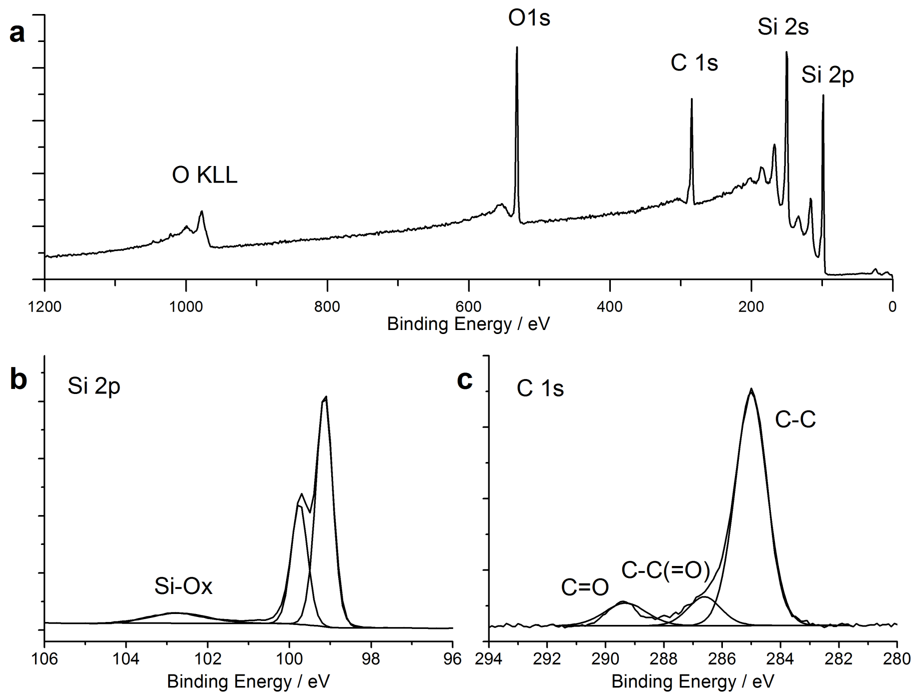

Figure S2. XPS spectra of silicon sample modified with undecylenic acid (control sample). a) Survey scan; b) high-resolution scan of the Si 2p region, showing a clear Si $2p_{1/2}$–Si $2p_{3/2}$ spin–orbit-split doublet (99.9 and 99.3 eV, respectively) from the bulk silicon and some silicon oxides in the range of 102-104 eV. The fractional monolayer coverage of oxidized silicon, calculated directly from the oxidized-to-bulk Si 2p peak area ratio according to a conventional model,[1] is approximately 0.5 monolayer equivalents. The silicon oxide is likely caused by the unreacted silicon hydride bonds underneath the monolayer being oxidized again when exposed to air. As Si(100) surface is terminated with a mixture of SiH, SiH_2 and SiH_3 species, the orientation of Si(100) may lead to the formation of self-assembled monolayers (SAMs) not as densely packed as that on Si(111). Several studies have shown that densely packed SAMs on silicon can prevent the oxidation of silicon more efficiently.[2-4] Therefore, it is easier for the unreacted silicon hydride on Si(100) to be oxidized. Nevertheless, forming an oxide-free surface on Si(100) after hydrosilylation is still possible as reported by other

researchers.[5-7]; c) C 1s narrow scan, revealing an asymmetric peak at 285.0 eV attributed to aliphatic C–C carbons, a second peak at 286.5 eV assigned to carbon adjacent to the carbonyl moiety (–C̲C(O)OH) and a peak at 290.0 eV for carbonyl –C̲(O)OH. Experimentally the peak area ratio of aliphatic C–C and two other peaks is 8 : 1 : 1, which is close to the value (9 : 1 : 1) predicted by the stoichiometry of the atoms of undecylenic acid.

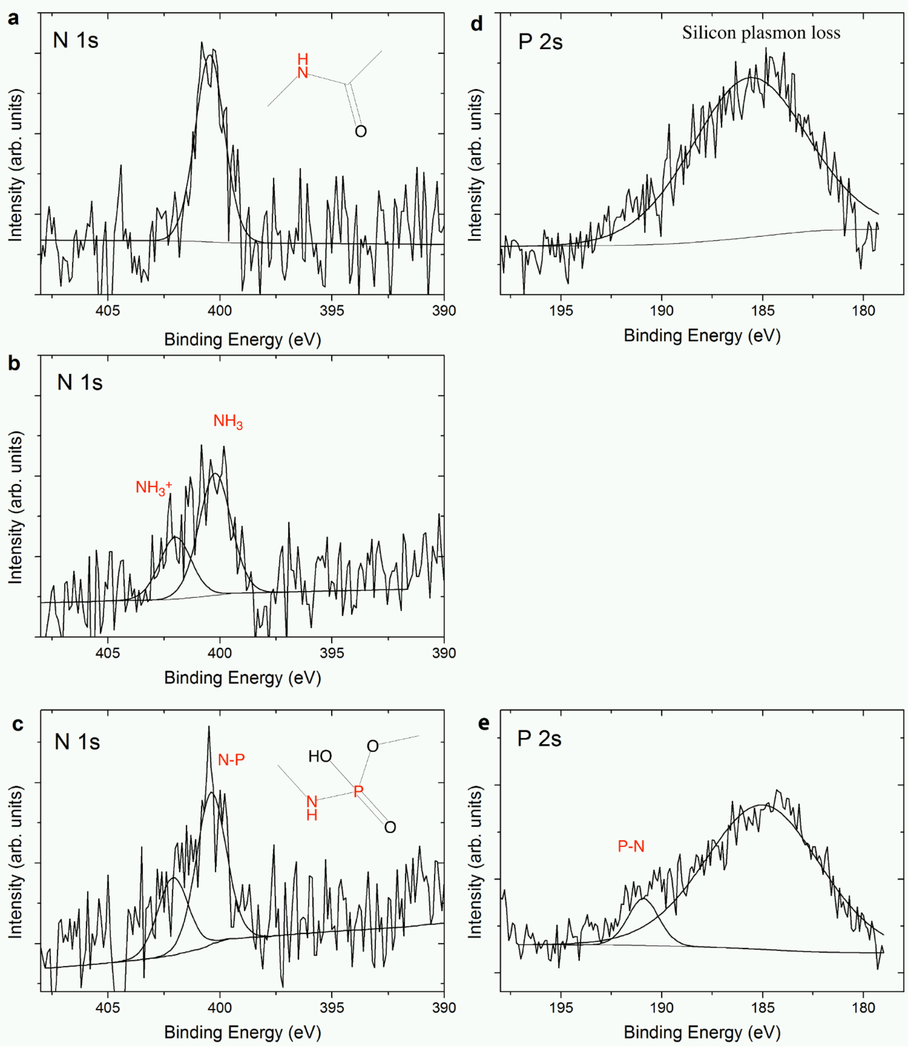

Figure S3. XPS spectra of chemically modified Si surfaces. a) The nitrogen 1s spectrum of the *N*-Boc-allylamine monolayer on silicon, showing a single peak at 400.5 eV. After treatment in 25% TFA (b) two peaks are recorded. The NH_3^+ and

the primary amine NH_2 have been assigned to the peaks at 402.0 eV and 400.2 eV, respectively. This indicates amine-terminated surface has been formed. The surface further reacted with phosphate shows a peak at 400.6 eV corresponding to newly formed N−P bonding and a peak at 402.0 eV suggesting the unreacted amine group on the surface (c). According to the ratio of two peak areas, the phosphate coupling efficiency can be estimated as 65%. Hence, the ratio of nitrogen atom and phosphorus atom on sample #2 is about 1.54. d) The phosphorus 2s spectra of the *N*-Boc-allylamine modified sample displays a broad peak at 185.0 eV, corresponding to silicon plasmon loss[8]. After the treatment of 25% TFA and phosphate, a new peak appears on the shoulder at 190.9 eV (e), assigned to the phosphorus from N−P bonding on the surface. Note that the peak area of N−P component in N 1s spectrum is equal to the peak area in P 2s spectrum. The above results, consistent with literature,[9] demonstrate the successful modification on silicon surfaces.

Van der Pauw measurement

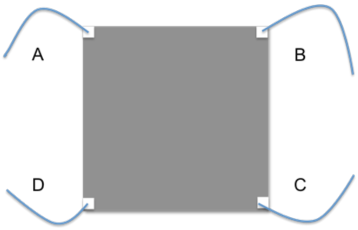

The shape of the samples for resistivity measurement is square with point ohmic electrodes contacted at the four corners. A voltage is applied to flow current I_{AB} along one side of the square sample and the voltage along the opposite side, V_{CD}, is measured. The current I_{AB} and voltage V_{CD} are then plotted to obtain the resistance for one side $R_{AB,CD}$. After repeating the process to the four sides of the square sample, the sheet resistance of the sample can be deduced from the following equations.

$$\exp\left(-\frac{\pi R_x}{R_s}\right)+\exp\left(-\frac{\pi R_y}{R_s}\right)=1 \quad (1)$$

$$R_x=\frac{1}{2}\left(R_{AB,CD}+R_{CD,AB}\right), R_y=\frac{1}{2}\left(R_{BC,AD}+R_{AD,BC}\right) \quad (2)$$

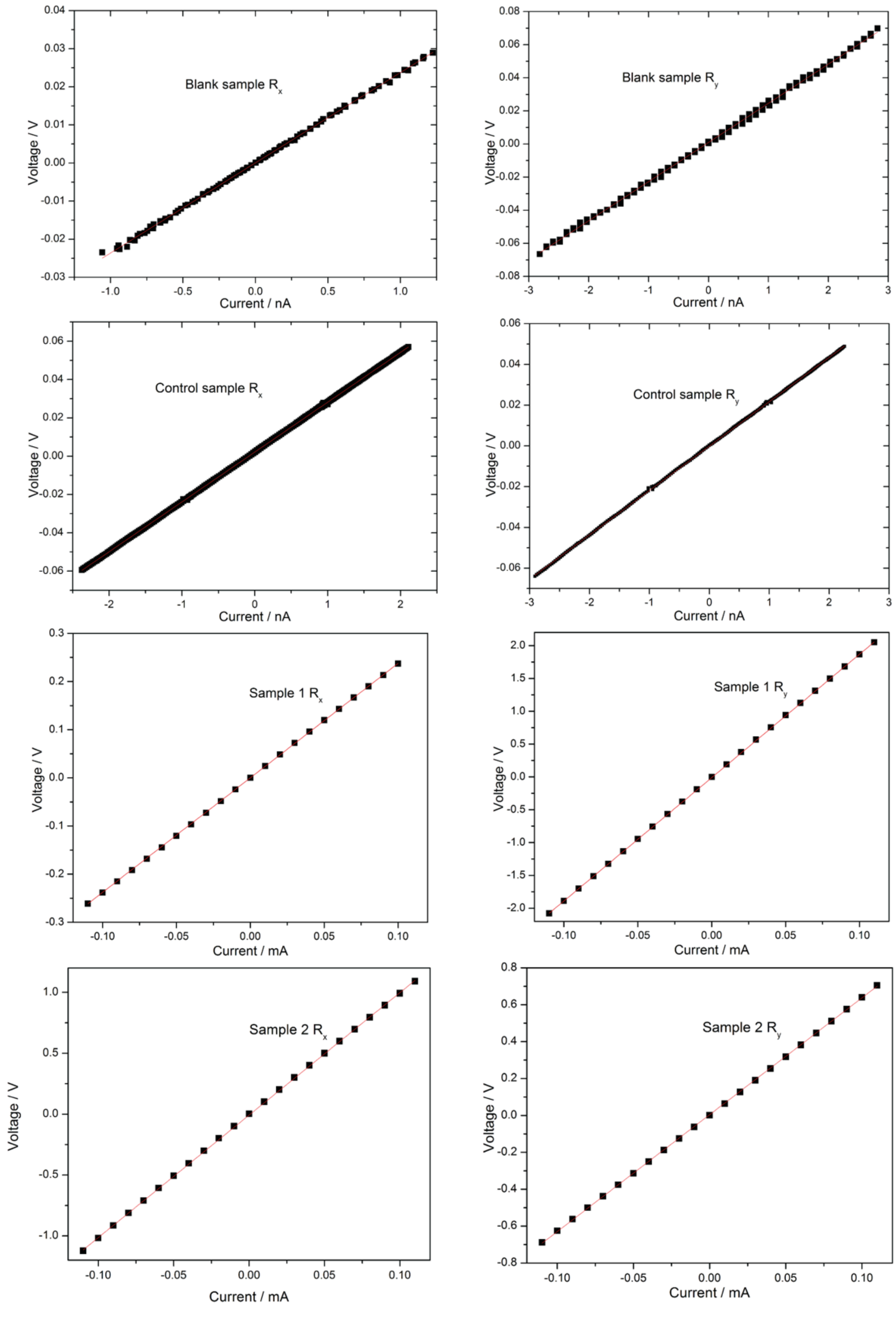

Figure S4. I-V curves from Van der Pauw measurements on blank, control samples, sample #1 and sample #2.

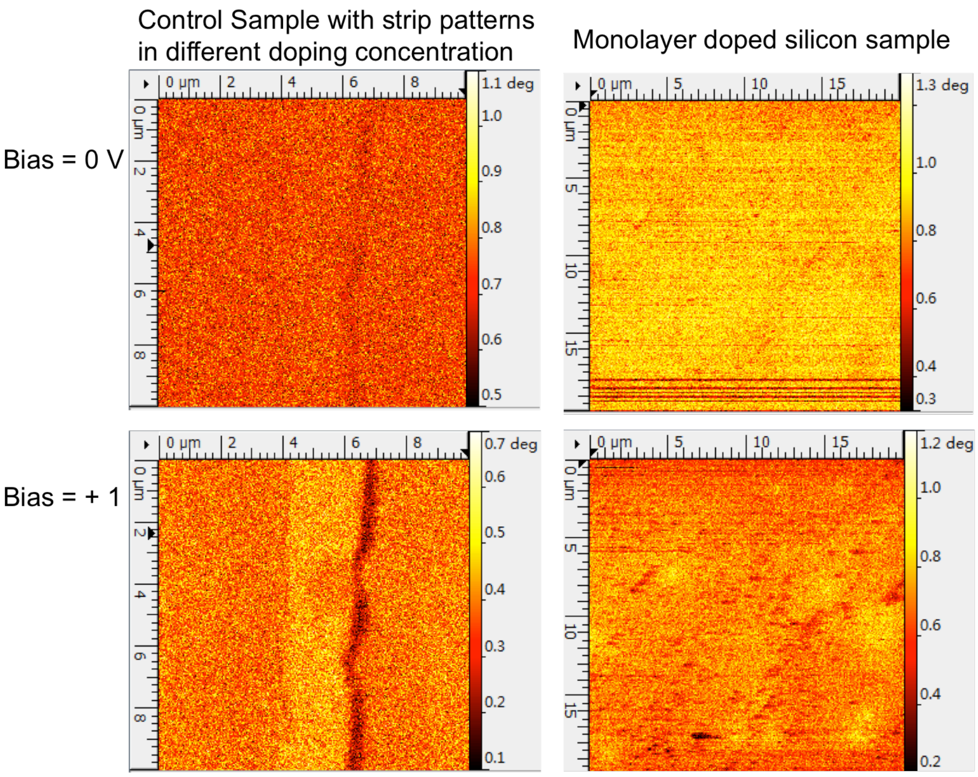

Figure S5. Electrostatic force microscope images on control (left) and monolayer nitrogen-doped samples (right). The top two images were recorded when no voltage was applied on the probe tip. The bottom images were taken at positive bias of 1 V. Under positive bias, the sample with different doping concentration patterns induced different static force towards the probe tip and thus rendered a phase shift profile. The image for nitrogen-doped sample with + 1 V bias shows no abrupt change in the profile, suggesting the even distribution of dopants.

Low-temperature Hall measurement

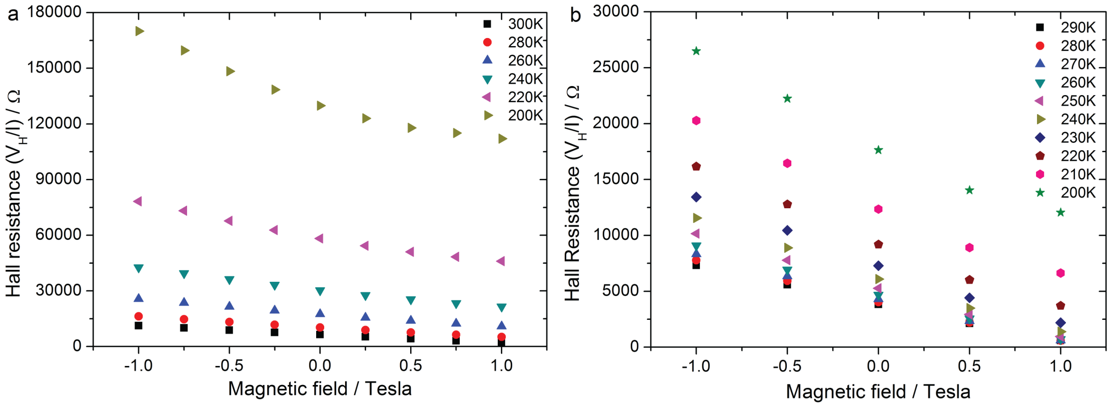

Figure S6. Dependence of the Hall resistance of the nitrogen-doped sample #1 (a) and both N and P doped sample #2 (b) on magnetic flux density at different temperature from 300 K to 200 K.

At low temperatures, the hall measurement results from sample #1 show non-linear correlation between measured resistance and magnetic field in Fig. S6. It was due to the magnetoresistance effect, as the resistance of a semiconductor generally increases when the sample is placed in a magnetic field. The measured resistance between point 1 and point 2 (R_{12}) in the Hall bar is not the actual Hall resistance (R_H), but the combination of magnetoresistance (R_M) and R_H. To extract the actual R_H and free carrier concentration at low temperature, the resistance between point 2 and point 3 (R_{23}) was also recorded. For no Hall effect should be detected between point 2 and point 3, R_{23} is consisted of its original resistance (R_S) and R_M. As an example, the experimental results of R_{23} and R_{12} in the function of magnetic field for sample #1 at 240 K are showed in Fig. S7.

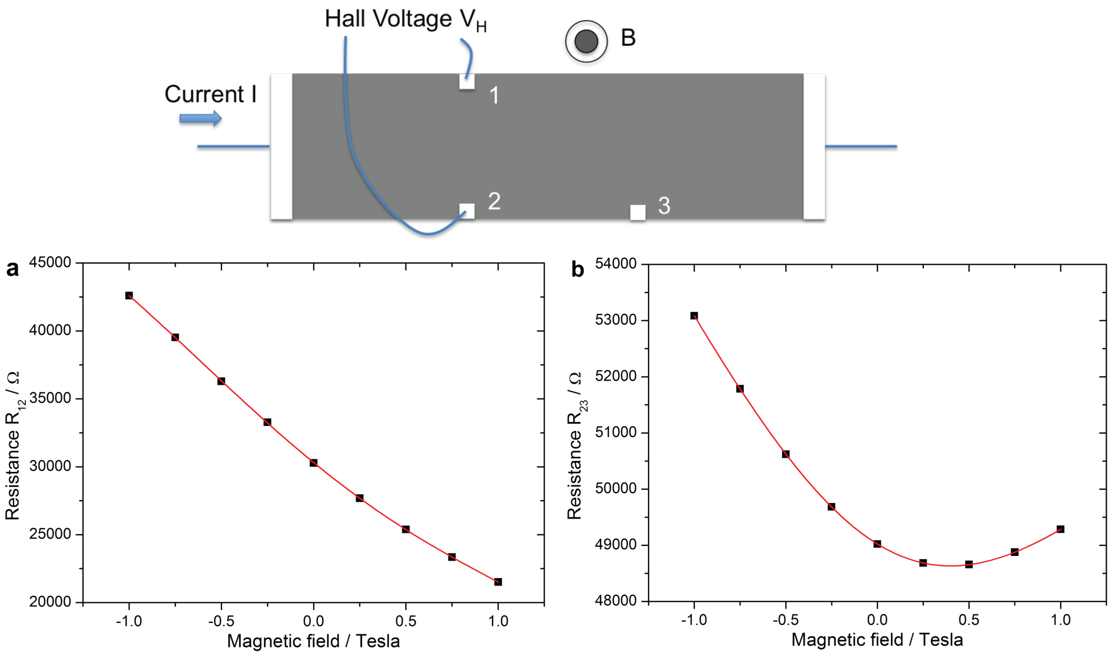

Figure S7. Hall resistance *Vs* Magnetoresistance. a) Dependence of resistance between point 1 and point 2 (R_{12}) on the change of magnetic field. b) Dependence of resistance between point 2 and point 3 (R_{23}) on magnetic field.

The two curves in Fig. S7 were then fitted with the following equations, respectively.

$$R_{12} = R_{M12} + R_H = r_{12}\left(a + cx^2 + ex^4 + gx^6 + \cdots\right) + r_H(bx + dx^3 + fx^5 + \cdots) \quad (3)$$

$$R_{23} = R_{M23} + R_S = r_{23}\left(a + cx^2 + ex^4 + gx^6 + \cdots\right) + r_S(bx + dx^3 + fx^5 + \cdots) \quad (4)$$

where x is the magnetic flux density and parameters such as a, b, c, d, e, f, g ... are the same ones in equation (3) as in equation (4). The fitting result of $r_H(bx + dx^3 + fx^5 + \cdots)$ is the actual Hall resistance. Hence, the free carrier concentration n_c can be obtained by the equation (5).

$$r_H b = -\frac{1}{n_c e}, n_c = -\frac{1}{r_H be} \quad (5)$$

Table S1. Free carrier concentration (n_c) for sample #1 and #2 at different temperature.

Sample #1 (nitrogen doping)		Sample #2 (both nitrogen and phosphorus)	
Temperature (K)	n_c (× 10^{11} cm^{-2})	Temperature (K)	n_c (× 10^{11} cm^{-2})
300	1.36	300	2.10
280	1.12	290	1.85
260	0.838	280	1.72
240	0.562	270	1.61
220	0.369	260	1.48
200	0.202	250	1.34
190	0.135	240	1.21
180	0.0790	230	1.09
170	0.0401	220	0.987
160	0.0155	210	0.898
		200	0.843

The donor energy level in nitrogen doped silicon

In the monolayer doped silicon samples, the doping is highly non-uniform and majority of the dopants are located within a thin layer below the surface. However, the electron concentration still follows the universal equation below to maintain charge neutral:

$$n_c = n_D^+ + p_c$$

where n_c is the average electron concentration, n_D^+ the average concentration of ionized dopants and p_c the average hole concentration.

For our case, the thin doped layer is always n-type, and hence $n_c >> p_c$. Then, we have

$$n_c = n_D^+$$

$$\rightarrow n_c = N_c \exp\left(\frac{E_F - E_c}{kT}\right) = \frac{N_D}{1 + 2\exp\left(\frac{E_F - E_d}{kT}\right)} = n_D^+ \quad \text{(a)}$$

$$\exp\left(\frac{E_F - E_c}{kT}\right) = \frac{n_c}{N_c}$$

where N_c is the effective density of states function, $N_c \approx 2\left(\frac{2\pi m_n^* kT}{h^2}\right)^{3/2} = w(kT)^{3/2}$

with w being the constant related to the band structure of the semiconductor, N_D the concentration of donors, E_F the Fermi level, E_c the conductance band edge and E_d the donor energy level.

Further, the right part of equation (a) can be written as:

$$\frac{N_D}{1+2\exp\left(\frac{E_F-E_d}{kT}\right)}=\frac{N_D}{1+2\exp\left(\frac{E_c-E_d}{kT}\right)\exp\left(\frac{E_F-E_c}{kT}\right)}$$

In the end, equation (a) can be rewritten as:

$$n_c=N_c\exp\left(\frac{E_F-E_c}{kT}\right)=\frac{N_D}{1+2\exp\left(\frac{E_c-E_d}{kT}\right)\frac{n_c}{N_c}} \quad \text{(b)}$$

After further simplifying equation (b) and solving the 2-order equation, we will find

$$n_c=\frac{-N_c+\sqrt{N_c^2+8N_cN_D\exp(\frac{\Delta E}{kT})}}{4\exp(\frac{\Delta E}{kT})} \quad \text{(c)}$$

where the activation energy $\Delta E = E_c - E_d$.

Sheet resistance results from a different type of wafer

Si (111) wafer as doping substrate was also employed into the nitrogen doping experiment. The sheet resistance results of the Si (111) sample before and after monolayer doping is shown in Table S2. The result in line with that of Si (100) also suggests a successfully nitrogen doping in Si (111) by self-assembled monolayer.

Table S2. Sheet resistances measured by Van der Pauw technique

Si (111) Samples with an original resistivity of 15 kΩ.cm, thickness of 300 μm	Sheet Resistance R_s (kΩ)
Blank sample: intrinsic silicon sample without any surface modification	457 ± 23
Sample #1: modified with monolayer containing nitrogen 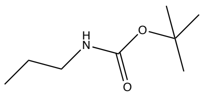	165 ± 27

Additional SIMS profiles for monolayer doped samples

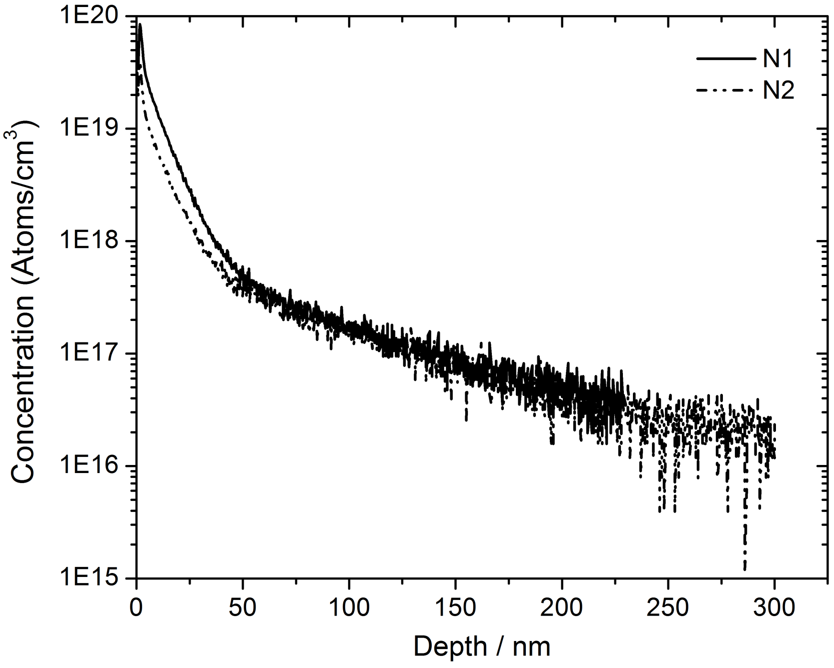

Figure S8. The duplicate nitrogen profiles of the N doped sample by SIMS, showing good reproducibility of the experimental results.

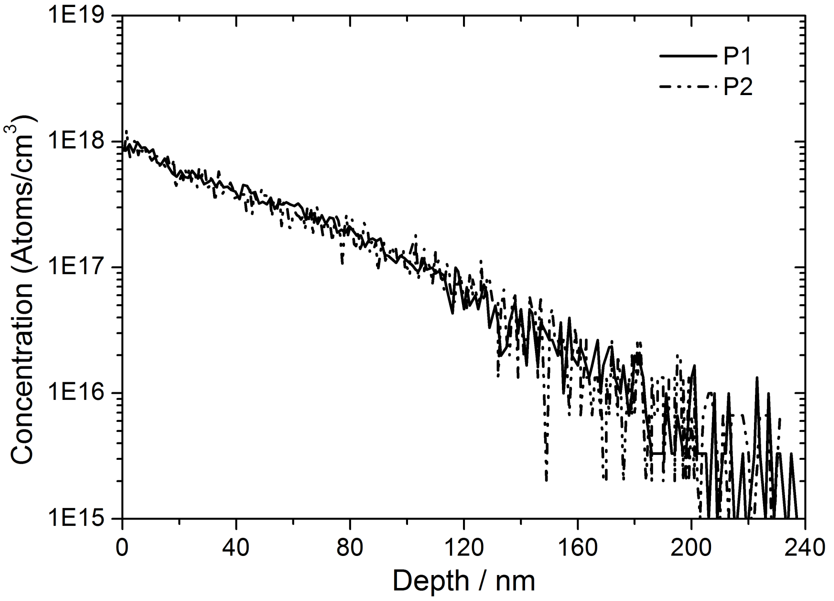

Figure S9. The duplicate phosphorus profiles of the N and P doped sample by SIMS.

Table S3. Comparison of SiO_2 formation methods[§]

Methods for SiO2 formation	ALD	Spin-on-glass (SOG)	Thermal evaporation
Silicon sample (measured sheet resistance of 143 ± 21 kΩ)	Sheet Resistance R_s (kΩ)		
Control: unmodified wafer coated with SiO_2 and annealed	121±13	116±6	109*
N-doped: modified with N monolayer, coated with SiO_2 and annealed	35.6±0.2	41.2±6.8	N.A.

[§] Only the data by ALD-SiO_2 capping are displayed in the manuscript, because the experimental data using SOG and thermal evaporation to form SiO_2 coating are incomplete due to non-scientific reasons.

* Only one repeat was done for thermally evaporated SiO_2.

References

1 Webb, L. J. & Lewis, N. S. Comparison of the Electrical Properties and Chemical Stability of Crystalline Silicon(111) Surfaces Alkylated Using Grignard Reagents or Olefins with Lewis Acid Catalysts. *J. Phys. Chem. B* **107**, 5404-5412,(2003).

2 Sieval, A. B., Linke, R., Zuilhof, H. & Sudholter, E. J. R. High-quality alkyl monolayers on silicon surfaces. *Adv. Mater.* **12**, 1457-1460,(2000).

3 Cicero, R. L., Linford, M. R. & Chidsey, C. E. D. Photoreactivity of Unsaturated Compounds with Hydrogen-Terminated Silicon(111). *Langmuir* **16**, 5688-5695,(2000).

4 Puniredd, S. R., Assad, O. & Haick, H. Highly stable organic monolayers for reacting silicon with further functionalities: the effect of the C− C Bond nearest the silicon surface. *J. Am. Chem. Soc.* **130**, 13727-13734,(2008).

5 Ciampi, S. *et al.* Functionalization of Acetylene-Terminated Monolayers on Si(100) Surfaces: A Click Chemistry Approach. *Langmuir* **23**, 9320-9329,(2007).

6 Lavi, A., Cohen, H., Bendikov, T., Vilan, A. & Cahen, D. Si-C-bound alkyl chains on oxide-free Si: towards versatile solution preparation of electronic transport quality monolayers. *Phys. Chem. Chem. Phys.* **13**, 1293-1296,(2011).

7 Peng, W., DeBenedetti, W. J. I., Kim, S., Hines, M. A. & Chabal, Y. J. Lowering the density of electronic defects on organic-functionalized Si(100) surfaces. *Appl. Phys. Lett.* **104**, 241601,(2014).

8 Gouzman, I., Dubey, M., Carolus, M. D., Schwartz, J. & Bernasek, S. L. Monolayer vs. multilayer self-assembled alkylphosphonate films: X-ray photoelectron spectroscopy studies. *Surf. Sci.* **600**, 773-781,(2006).

9 Strother, T., Hamers, R. J. & Smith, L. M. Covalent attachment of oligodeoxyribonucleotides to amine-modified Si (001) surfaces. *Nucleic Acids Res.* **28**, 3535-3541,(2000).
